# 射频透热联合化疗治疗肺癌恶性心包积液的近期疗效

**DOI:** 10.3779/j.issn.1009-3419.2011.07.06

**Published:** 2011-07-20

**Authors:** 鹏飞 罗, 培国 曹, 志平 姚

**Affiliations:** 410013 长沙，中南大学湘雅三医院肿瘤科 Department of Medical Oncology, the Tird Xiangya Hospital of Central South University, Changsha 410013, China

**Keywords:** 热疗, 肺肿瘤, 化疗, 恶性心包积液, 生活质量, Hyperthermia, Lung neoplasms, Chemotherapy, Malignant pericardial effusion, Quality of life

## Abstract

**背景与目的:**

恶性心包积液为肺癌严重并发症之一，有效治疗方法不多。本研究旨在评价射频透热联合化疗治疗恶性心包积液的近期疗效。

**方法:**

回顾性分析中南大学湘雅三医院肿瘤科2000年10月-2010年10月收治的肺癌并恶性心包积液的患者55例，分为射频透热联合化疗组（热化组）和化疗组，热化组采用心包穿刺引流心包积液后，给予心包腔内注射顺铂20 mg+地塞米松5 mg，然后进行局部射频透热治疗，患者一般情况改善后行全身化疗，腔内化疗1次-6次，平均3次，热疗每周2次，平均6次，腔内温度控制在40.5 oC-41.5 oC左右并维持60 min；化疗组只给予腔内注药和全身化疗。全身化疗方案为长春瑞滨+顺铂，长春瑞滨总量50 mg/m^2^，顺铂总量75 mg/m^2^。

**结果:**

热化组心包积液完全缓解率为54.3%，总有效率为91.4%，化疗组分别为25.0%、70.0%，两组比较有统计学差异（*P* < 0.05）；治疗后两组生活质量均明显提高，但热化组生活质量的改善优于化疗组，两组KPS评分的变化有统计学差异（*P* < 0.05）；化疗不良反应主要为消化道毒性和骨髓抑制，两组比较无统计学差异（*P*>0.05）；与热疗相关的主要副反应为局部皮肤疼痛（8.6%）和皮下脂肪硬结（5.7%）。

**结论:**

射频透热联合化疗治疗肺癌恶性心包积液近期疗效确切，能显著改善患者生活质量，不增加全身化疗毒副反应，安全性高。

## Abstract (en)

**Background and objective:**

Malignant pericardial effusion is one of the serious complications of lung cancer and lack effective treatment methods. The aim of this study is to evaluate the efficacy and safety of radiofrequency hyperthermia combined with chemotherapy for patients with malignant pericardial effusion caused by lung cancer.

**Methods:**

Fify-five patients with malignant pericardial effusion caused by lung cancer were divided into hyperthermia combined with chemotherapy group (combined therapy group) and chemotherapy group. The combined therapy group was treated with radiofrequency hyperthermia afer the pericardiocentesis and intracavitary injection (cisplatin 20 mg and dexamethasone 5 mg), when patients' general state of health improved, systemic chemotherapy was performed. The chemotherapy group was treated only with intracavitary injection and systemic chemotherapy. Intracavitary chemotherapy was performed for 1-6 times (average 3 times). Hyperthermia was performed twice per week with an average of 6 times following intracavitary and systemic chemotherapy. The temperature of intracavitary was 40.5 oC-41.5 oC for 60 min during the hyperthermia periods. Systemic chemotherapy consists of cisplatin (75 mg/m^2^) and vinorelbine (50 mg/m^2^).

**Results:**

The complete remission rate (CR) of malignant pericardial effusion was 54.3% and the response rate (RR) was 91.4% in the combined therapy group, while the rates of CR and RR of chemotherapy group were 25.0% and 70.0%, and the differences of CR and RR between the two groups were signifcant (*P* < 0.05). Afer treatment, the quality of life improved signifcantly in both groups, but the combined therapy group had a higher KPS score than in the chemotherapy group (*P* < 0.05). The adverse events associated with the chemotherapy included gastrointestinal toxicity and myelosup-pression, and there were no signifcant differences between the two groups. The main side effects associated with radiofrequency hyperthermia included local skin ache (8.6%) and induration of subcutaneous fat (5.7%).

**Conclusion:**

Radiofrequency hyperthermia combined with chemotherapy is effective in treatment of malignant pericardial effusion and can signifcantly improve the quality of life of patients without increasing the toxicity of chemotherapy, and side effects could be tolerable.

恶性心包积液是晚期肺癌患者常见并发症之一，严重影响患者的生活质量，心包内药物注射是治疗恶性心包积液最常用的方法之一，但存在疗效不稳定、药物毒副反应大等问题。卞宝祥等^[1]^对27例肺癌所致心包积液患者采用心包置管持续引流并注人化疗药物治疗，近期总有效率仅为70.4%。有研究^[2, 3]^表明，热疗与化疗有协同抗肿瘤机制，在治疗恶性体腔积液时疗效显著^[4]^，但目前临床上关于热化综合治疗恶性心包积液的研究并不多见。本研究回顾性分析中南大学湘雅三医院肿瘤科2000年10月-2010年10月收治的肺癌并恶性心包积液的患者55例，其中35例采用射频透热联合化疗治疗，取得了较好的近期疗效，现报告如下。

## 1 资料与方法

### 1.1 一般资料

全组共55例，均经病理学或细胞学确诊为原发性支气管肺癌并恶性心包积液，其中腺癌41例、鳞癌11例、小细胞肺癌3例。按治疗方法不同，分为射频透热联合化疗组（热化组）35例和化疗组20例。热化组中男性26例，女性9例，年龄47岁-73岁，平均年龄62.5±5.8岁；化疗组中男性14例，女性6例，年龄42岁-72岁，平均年龄63.2±6.6岁。治疗前患者均伴有呼吸困难、心动过速、低电压、心音低远、心界扩大、颈静脉怒张、静脉压增高等症状及体征，心脏B超示舒张期心包腔液性暗区在20 mm以上。两组病例接受治疗前在性别、年龄、病理类型、卡式评分（Karnofsky performance status, KPS）等方面均具可比性（表 1）。

**1:** 热化组与化疗组临床资料比较 Comparisons of clinical characteristics between radiofrequency hyperthermia combined with chemotherapy group and chemotherapy group

Characteristic	Radiofrequency hyperthermia combined with chemotherapy group (*n* =35)	Chemotherapy group (*n* =20)	*χ*^2^	*P*
Gender			0.118	0.731
Male	26	14		
Female	9	6		
Age (year)			1.920^*^	0.055
Range	47-73	42-72		
Mean±SD	62.5±5.8	63.2±6.6		
Pathological type			0.013	0.994
Adenocarcinoma	26	15		
Squamous cell carcinoma	7	4		
Small cell lung cancer	2	1		
KPS score			0.365	0.947
30-40	4	2		
40-50	14	7		
50-60	9	5		
60-70	8	6		
KPS: Karnofsky performance status; ^*^: *t* test.				

### 1.2 治疗方法

#### 1.2.1 引流加胸腔注药

所有患者经B超定位后行心包穿刺术，采用心包腔闭式引流，尽可能引流心包积液，但应控制排放速度，日排放量不超过500 mL，避免影响患者的心、肺功能。引流心包积液后向腔内注入顺铂20 mg，另加地塞米松5 mg腔内注射以减轻化疗药物的局部刺激反应，注药后嘱患者变换体位，以便化疗药物均匀分布于心包腔内，腔内化疗1次-6次，平均3次，热化组患者每次腔内化疗后行射频透热治疗。

#### 1.2.2 射频透热

采用深圳先科公司生产的SR-1000型射频热疗机进行，电容式加热，频率40.68 MHz，极板直径20 cm-30 cm，加热时极板置于心前区，入射功率400 W-900 W，反射功率控制在3%以内，每次治疗时间70 min-90 min，温度稳定在40.5 ℃-41.5 ℃维持60 min，每周2次，治疗次数2次-20次，平均6次。

#### 1.2.3 测温方法

将热电偶测温探头通过心包引流管进入心包腔，测温探头顶端以不超过心包引流管最前端开口处为宜，在心包腔温度达41 ℃时持续加温。

#### 1.2.4 静脉化疗

两组患者心包积液得到初步控制，患者一般情况改善后即开始全身静脉化疗，化疗方案为顺铂加长春瑞滨，顺铂腔内加静脉总量为75 mg/m^2^，长春瑞滨总量50 mg/m^2^，热化组患者每次静脉化疗后即行射频透热治疗。

### 1.3 疗效评定

心包积液疗效判断：根据胸腔CT、胸片、心包B超等影像学诊断参考WHO标准^[5]^判定近期疗效，分为完全缓解（complete response, CR）：体液完全吸收持续4周以上；部分缓解（partial response, PR）：积液吸收5 0%以上，持续4周以上；无变化（stable disease, SD）：积液减少不足50%，增加不足25%；进展（progressive disease, PD）：积液增加大于25%。生活质量：按KPS评分标准^[6]^在治疗前、治疗后4周评定。好转：KPS评分增加≥10分，并维持4周以上；稳定：KPS评分无明显变化；恶化：KPS评分减少≥10分。化疗毒副反应：按WHO规定标准分级^[7]^，化疗前后各查1次血常规、肝肾功能，化疗期间每周查1次血常规。

### 1.4 统计学处理

采用SPSS 17.0统计软件处理，计数资料以率表示，采用*χ*^2^检验，计量资料如服从正态分布，以Mean±SD表示，采用*t*检验，*P* < 0.05为有统计学差异。

## 2 结果

### 2.1 心包积液疗效

热化组完全缓解率（CR）为54.3%，总有效率（CR+PR）为91.4%，化疗组分别为25.0%和70.0%，热化组近期疗效明显高于化疗组，差异有统计学意义（*P* < 0.05）（表 2）。

**2:** 热化组与化疗组患者心包积液近期疗效比较 Comparisons of the response rate of malignant pericardial effusion between radiofrequency hyperthermia combined with chemotherapy group and chemotherapy group

Group	*n*	CR	PR	SD	PD	CR+PR
Radiofrequency hyperthermia combined with chemotherapy group	35	19 (54.3%)	13 (37.1%)	3 (8.6%)	0 (0)	32 (91.4%)
Chemotherapy group	20	5 (25.0%)	9 (45.0%)	3 (15.0%)	3 (15.0%)	14 (70.0%)
*χ*^2^		4.438				4.270
*P*		0.035				0.039
CR: complete response; PR: partial response; SD: stable disease; PD: progressive disease.						

### 2.2 生活质量变化

两组治疗前后KPS评分比较均有统计学差异（*P* < 0.05），治疗后大部分患者呼吸困难、心动过速、低电压、心音低远、心界扩大、颈静脉怒张、静脉压增高等症状及体征好转，生活质量明显提高。但热化组生活质量的改善优于化疗组，两组KPS评分变化有统计学差异（*P* < 0.05）（表 3）。

**3:** 热化组与化疗组治疗前后患者KPS评分比较 Comparisons of KPS scores between radiofrequency hyperthermia combined with chemotherapy group and chemotherapy group before and after treatment

Group	*n*	Before treatment	After treatment	Change of KPS score
Radiofrequency hyperthermia combined with chemotherapy group	35	52.8±9.5	71.3±9.5^*^	19.6±5.0^▲^
Chemotherapy group	20	53.6±9.6	62.3±11.8^#^	10.2±6.7
^*^: Compared with before treatment of radiofrequency hyperthermia combined with chemotherapy group, *t*=65.591, *P* < 0.001; ^#^: Compared with before treatment of chemotherapy group, *t*=23.868, *P* < 0.001; ^▲^Compared with chemotherapy group, *t*=38.928, *P* < 0.001.				

### 2.3 化疗不良反应

主要为消化道毒性与骨髓抑制，均为Ⅰ度-Ⅱ度，消化道毒性表现为恶性呕吐、腹泻、便秘，骨髓抑制表现为白细胞下降、血小板减少、贫血，两组比较差异无统计学意义（*P* > 0.05）（表 4）。

**4:** 热化组与化疗组不良反应比较 Chemotherapy-related adverse events between radiofrequency hyperthermia combined with chemotherapy group and chemotherapy group

Adverse event	Radiofrequenc y hyper thermia combined with chemotherapy group (*n* =35) [*n*/%]	Chemotherapy group (*n* =20) [*n*/%]	*χ*^2^	*P*
Leukopenia	5 (14.3)	4 (20.0)	0.304	0.582
Thrombocytopenia	2 (5.7)	2 (10.0)	0.347	0.556
Anemia	4 (11.4)	3 (15.0)	0.146	0.702
Nausea or vomiting	9 (25.7)	6 (30.0)	0.118	0.731
Diarrhea	4 (11.4)	2 (10.0)	0.027	0.870
Constipation	7 (20.0)	5 (25.0)	0.187	0.666

### 2.4 与热疗相关的不良反应

在射频透热治疗中，多数患者有出汗症状，但无临床不适，正常饮食者无须补液纠正，一般情况差者适当补液处理，防止电解质紊乱。局部皮肤疼痛3例（8.6%），皮下脂肪硬结2例（5.7%），未经特殊处理能自行缓解。为判断局部射频透热是否存在心脏损害，热疗前后行心肌酶学检查，主要检测指标为CK、CK-MB和α-HBDH。热化组35例患者行射频透热治疗前后心肌酶学水平无统计学差异（*P* > 0.05）（表 5）。

**5:** 热化组热疗前后心肌酶指标比较 Comparisons of myocardial enzymes level before and after hyperthermia in the combind therapy group

Indicator	Before hyperthermia (U/L)	After hyperthermia (U/L)	*t*	*P*
CK	136.2±53.6	132.6±48.3	1.658	0.097
CK-MB	14.0±4.3	13.4±4.8	1.952	0.051
α-HBDH	150.8±39.4	148.8±45.7	1.932	0.053

## 3 讨论

肺癌恶性心包积液多通过原发肿瘤经血、淋巴道转移或直接侵犯心包所致。恶性心包积液一旦出现，进展迅速，液体大量积聚心包腔内而出现心包填塞，使心包内压力升高，达到一定程度后，心室舒张功能受限，心搏出量下降，肺循环和体循环阻力均升高，血流动力学变慢，表现为呼吸困难、心音低钝遥远、奇脉等症状，严重影响了患者的生活质量和生存期，如不及时治疗很可能会导致患者死亡^[8]^。

恶性心包积液的治疗，临床上目前多以B超引导下穿刺放液及腔内注射抗肿瘤药物的局部治疗为主，虽有一定疗效，却存在疗效不稳定、患者缓解率低、生活质量改善不明显等问题。研究^[4, 9]^表明，热效应能使癌细胞结构破坏，且催化药物与癌细胞DNA的加成反应，抑制DNA修复和多药耐药性p-糖蛋白的表达，提高化疗疗效。顺铂是目前心包腔内局部化疗的常用药物之一，具有分子量大、水溶性、渗透力强等特点。顺铂属重金属复合物，稀释后稳定，它能直接破坏DNA结构，注入腔内后不易透过心包腔屏障，使心包腔内浓度远高于血浆内浓度，并可渗入到肿瘤表面一定厚度，同时有研究^[10, 11]^表明，顺铂能随着温度增加而提高杀伤肿瘤细胞的作用，这为我们进行局部热疗联合化疗治疗恶性心包积液提供了基本依据。

目前临床上关于热疗联合化疗治疗恶性胸腹水的报道较多，部分研究资料显示疗效明显^[12, 13]^。但关于热疗联合化疗治疗恶性心包积液的报道较少，国内孙静等^[14]^采用B超定位下放置心包腔内引流管，随后将溶有化疗药物的生理盐水在体外加温至45 ℃，经引流管缓慢滴入心包腔，反复冲洗至引流液无色，但热灌注治疗是在体外加热液体，操作过程中液体温度难以精确控制，且温度波动范围较大，这就给治疗过程中确保疗效及患者的安全带来了一定的困难。

射频透热是近年来开展的治疗恶性肿瘤的新技术，其治疗原理是通过容性射频电磁场的辐射作用使机体局部区域内的电解质离子、分子发生快速移动，振荡而产热。国内刘跃等^[15]^采用射频透热治疗配合常规放疗治疗癌性心包积液，利用电容式HG-2000型体外射频热疗机，加热靶区温度至42 ℃-43 ℃，治疗时间30 min-50 min，每周2次-3次，治疗8次-12次，中位次数10次，结果显示热放综合治疗组在近期疗效及中位生存期方面明显优于单纯放疗组，且射频透热治疗对心脏无毒副作用。我们在利用SR-1000型射频热疗机治疗恶性心包积液时，通过测温探头对心包腔内温度进行精确控制，使心包内温度稳定在40.5 ℃-41.5 ℃，既可以避免温度过高产生的心脏损害，也使肿瘤靶区达到持续恒定加温的目的。从本组资料看，热化组治疗恶性心包积液近期有效率为91.4%，明显高于化疗组（*P* < 0.05）；KPS评分提示热化组生活质量的改善优于化疗组（*P* < 0.0 5）；热化组全身化疗的毒性反应与化疗组之间无明显差别（*P* < 0.05）；在射频透热治疗期间，患者无明显心肌损害表现，仅少数患者出现局部皮肤疼痛（3例）和皮下脂肪硬结（2例），未经特殊处理均自行缓解。这提示射频透热联合化疗较单纯化疗在治疗肺癌恶性心包积液时有明显的优势，且安全性高，并为进一步的抗肿瘤综合治疗创造机会。关于射频透热联合化疗对肺癌恶性心包积液患者总生存期是否有益还有待进一步观察与研究。

## References

[1] Bian BX, Song ZY, Yang CX (2007). Clinical observation of pericardial injections in the treatment of pericardial effusion in patients with lung cancer. J Basic Clin Oncol.

[2] Issels RD (2008). Hyperthermia adds to chemotherapy. Eur J Cancer.

[3] Huang P, Zhou JY (2010). The enhancement of hyperthermia study on patient with tumor. Mod Oncol.

[4] Ding ZY, Xie XD (2010). Mechanisms and clinical application of hyperthermia for tumors. J Int Oncol.

[5] Patz EF Jr, McAdams HP, Goodman PC (1996). Ambulatory sclerotherapy for malignant pleural effusions. Radiology.

[b6] 6Yin WB, Yu ZH, Xu GZ, et al. Radiation oncology. 4th ed. Beijing: Peking Union Medical College Press, 2007. 1349.殷蔚伯, 余子豪, 徐国镇, 等主编. 肿瘤放射治疗学. 第四版. 北京: 中国协和医科大学出版社, 2007. 1349.

[b7] 7Zhou JC ed. Practice of medical oncology. 2nd ed. Beijing: People's Medical Publishing House, 2003. 29.周际昌主编. 实用肿瘤内科学. 第2版. 北京: 人民卫生出版社, 2003. 29.

[8] Wu YF, Yu L, Wang JW (2007). Advances in medical treatment of malignant pericardial effusion. Oncol Prog.

[9] Issels RD, Lindner LH, Verweij J (2010). Neo-adjuvant chemotherapy alone or with regional hyperthermia for localised high-risk soft-tissue sarcoma: a randomised phase 3 multicentre study. Lancet Oncol.

[10] Kampinga HH (2006). Cell biological effects of hyperthermia alone or combined with radiation or drugs: a short introduction to newcomers in the feld. Int J Hyperthermia.

[11] Yang XH, Fang MZ, Xu W (2008). A clinical study of thermotherapy combined intravenous chemotherapy and intraperitoneal chemotherapy for the treatment of advanced gastrointestinal carcinoma. Mod Oncol.

[12] Yin J, Dai P, Xie ZQ (2007). Clinical study of chemotherapeutic hyperthermic intraperitoneal perfusion combined with high frequency hyperthermia for the treatment of malignant ascites. Med J Wuhan Univ (Med).

[13] Cao D, Hou M, Gou HF (2006). Hyperthermia combined with intracavitary injection of drug for malignant pleural effusion. Chin J Lung Cancer.

[14] Sun J, Sheng ZJ (2005). Curative effect of lifein plus hyperthermic chemotherapy in treatment of malignant pericardial effusion. Chin J Clin Oncol Rehabil.

[15] Liu Y, Zhang GQ, Wang YG (2009). Clinical analysis of combined hyperthermia and radiotherapy for patients with malignant pericardial effusion. Mod Oncol.

